# 24-h NIHSS score is the strongest prognostic predictor of 90-day outcome in cardioembolic stroke patients with anterior circulation occlusion after endovascular thrombectomy

**DOI:** 10.3389/fneur.2026.1821974

**Published:** 2026-05-13

**Authors:** Chen Yang, Yangxi Chen, Qingyan Liu, Ge Gong, Lei Guo, Yu Liu, Xinghu Zhang

**Affiliations:** 1 Department of Geriatrics, Nanjing Jinling Hospital, Affiliated Hospital of Medical School, Nanjing University, Nanjing, Jiangsu, China; 2 Department of Geriatrics, Nanjing Jinling Hospital, Affiliated Hospital of Medical School, Southern Medical University, Nanjing, Jiangsu, China; 3 International Medical Services and VIP Medical Services, Binzhou People's Hospital, Binzhou, Shandong, China

**Keywords:** cardioembolic stroke, endovascular thrombectomy, NIHSS score, predictor, unfavorable functional outcome

## Abstract

**Background:**

This study aimed to evaluate and compare the predictive performance of the National Institutes of Health Stroke Scale (NIHSS) assessed at baseline, at 24 h, and derived change metrics for 90-day unfavorable functional outcome (modified Rankin Scale 3–6) in cardioembolic stroke patients with Anterior Circulation Occlusion (ACO) post-endovascular thrombectomy (EVT).

**Methods:**

A retrospective analysis of 103 eligible patients was performed. Univariate and multivariate logistic regression identified predictors. Receiver operating characteristic (ROC) curve analysis and DeLong’s test compared the predictive performance of baseline NIHSS, 24-h NIHSS, ΔNIHSS (baseline NIHSS − 24-h NIHSS) and the percent ΔNIHSS (ΔNIHSS × 100/baseline NIHSS).

**Results:**

Multivariate analysis confirmed 24-h NIHSS, baseline NIHSS, ΔNIHSS, and percent ΔNIHSS as independent predictors. ROC analysis showed that 24-h NIHSS had the highest predictive power (AUC = 0.850), significantly outperforming baseline NIHSS (AUC = 0.702), ΔNIHSS (AUC = 0.735), and percent ΔNIHSS (AUC = 0.780). The optimal cut-off value was ≥12, with 82.2% sensitivity and 75.6% specificity. Combining 24-h NIHSS with other NIHSS-based metrics did not improve predictive performance compared to 24-h NIHSS alone.

**Conclusion:**

The 24-h NIHSS score is the strongest prognostic predictor of 90-day unfavorable functional outcome in cardioembolic stroke patients post-EVT, superior to baseline scores, ΔNIHSS and percent ΔNIHSS. It serves as an early and effective tool for prognostic stratification.

## Introduction

Cardioembolic stroke is a common etiology of acute ischemic stroke, often associated with greater severity, worse prognosis, higher recurrence rates, and increased mortality (1). Endovascular therapy (EVT) is a well-established treatment for acute ischemic stroke. Although recanalization rates approach 80%, approximately 50% of patients still experience unfavorable functional outcome (modified Rankin Scale [mRS] ≥ 3) at 3 months (2). Therefore, early and accurate identification of patients at high risk of unfavorable functional outcome is crucial for guiding clinical decision-making, optimizing resource allocation, and setting realistic rehabilitation goals (3).

The National Institutes of Health Stroke Scale (NIHSS) is a gold standard for assessing stroke severity and prognosis. Baseline NIHSS and its early changes within 24 h have been established as strong predictors of long-term functional outcome (4–6). While the baseline NIHSS reflects initial stroke severity, early neurological changes within 24 h may not fully capture the effects of post-EVT recanalization, medical treatment, nursing care, and other interventions on outcome. The 24-h NIHSS score, by integrating both baseline stroke severity and early therapeutic response, may provide more robust prognostic value (7). Although studies have explored the predictive value of 24-h NIHSS in stroke patients (8–10), evidence specifically within the homogeneous population of Cardiogenic stroke with Anterior Circulation Occlusion (ACO) remains insufficient. Furthermore, whether its predictive performance is superior to baseline NIHSS or other metrics of early neurological change (ΔNIHSS and percent ΔNIHSS) remains unclear. This retrospective study aimed to evaluate the predictive value of the 24-h NIHSS score for 90-day functional outcome in cardioembolic stroke patients with ACO after EVT, and to systematically compare its performance with baseline NIHSS, ΔNIHSS, and percent ΔNIHSS.

## Methods

### Study design and population

This study included consecutive cardioembolic stroke patients with Anterior Circulation Occlusion (ACO) who underwent EVT at Jinling Hospital from March 2019 to February 2024 and achieved successful vascular recanalization. The study protocol was approved by the Ethics Committee of Jinling Hospital, Affiliated Hospital of Nanjing University Medical School (Approval No. 2010ly18). Since the study involved only the analysis of anonymized data without additional interventions and posed no potential risks to patients, the ethics committee waived the requirement for obtaining individual patient informed consent.

Inclusion criteria were as follows:

1. Age ≥ 18 years.
2. Cardioembolic stroke defined according to both the TOAST classification criteria (11) and the Chinese Expert Consensus on the Diagnosis of Cardioembolic Stroke (12). The TOAST classification was used for etiological categorization, while the 2020 consensus provided operational diagnostic criteria. According to the consensus, all included patients met the “definite” criteria for cardioembolic stroke, requiring the following:

- Necessary conditions:

- Typical clinical manifestations of acute ischemic stroke;
- Specific changes on head CT and/or MRI consistent with embolic infarction.

- Supporting conditions (at least one of the following):

- Cardioembolic source detected on cardiac ultrasound (e.g., left atrial/ventricular thrombus, valvular vegetation);
- Arrhythmia (especially atrial fibrillation) documented on electrocardiogram or Holter monitoring;
- Characteristic vascular imaging findings: sudden interruption of a major intracranial artery or its branch without evidence of significant atherosclerotic stenosis in the upstream vessels.

- Exclusion of other stroke etiologies (e.g., large-artery atherosclerosis, small vessel disease, other determined causes).

1. EVT performed within 24 h of symptom onset.
2. Post-procedural modified Thrombolysis in Cerebral Infarction (mTICI) grade of 2b–3 (13).

Exclusion criteria were: (1) pre-existing severe neurological disability (pre-stroke mRS > 2); (2) intracranial hemorrhage (ICH) before EVT; (3) severe liver/kidney disease, hematological disorders, or malignancy; (4) severe psychiatric illness or cognitive impairment; (5) incomplete clinical data.

### Data collection

General clinical data collected included: age, sex, smoking history, alcohol consumption, medical history, and Alberta Stroke Program Early CT Score (CT-ASPECTS) (14) on admission. Peri-procedural data included: onset-to-puncture time (OPT), puncture-to-reperfusion time (PRT), number of thrombectomy attempts, American Society of Interventional and Therapeutic Neuroradiology/Society of Interventional Radiology (ASTIN/SIR) score (15), and post-procedural mTICI score (16). Other assessments included: pre-stroke mRS (17), baseline NIHSS (17), NIHSS at 24 h after EVT, absolute change in NIHSS from baseline to 24 h (ΔNIHSS, calculated as baseline NIHSS − 24-h NIHSS) (18), and percent ΔNIHSS ([ΔNIHSS/baseline NIHSS] × 100) (18). For patients presenting within 4.5 h and eligible for intravenous thrombolysis, standard-dose recombinant tissue plasminogen activator (rt-PA) was administered prior to or during EVT. Endovascular thrombectomy included mechanical thrombectomy, intra-arterial thrombolysis, balloon angioplasty, stent placement, and/or intra-arterial tirofiban administration, used alone or in combination based on the specific condition. All patients underwent head CT within 24 h post-EVT to assess for ICH (19). ICH was defined as any intracranial hemorrhage (radiologically confirmed hemorrhage in any intracranial compartment).

### Outcome assessment

Clinical outcome were assessed at 90-day by neurologists via telephone or outpatient follow-up, blinded to the study treatment details. A favorable functional outcome was defined as mRS 0–2, and an unfavorable functional outcome as mRS 3–6 (20).

### Statistical analysis

Statistical analyses were performed using SPSS 22.0. Continuous variables were compared using *t*-tests or Mann–Whitney *U* tests, and categorical variables using chi-square tests. Variables with *p* < 0.05 in the univariate analysis were further incorporated into logistic regression for multivariate analysis to identify independent predictors. Predictive performance was evaluated using ROC curve analysis, and area under the curve (AUC) values were compared using DeLong’s test. A two-sided *p*-value < 0.05 was considered statistically significant.

## Results

A total of 103 cardioembolic stroke patients with ACO were included, Patients with a favorable functional outcome at 90-day after EVT were 41, while those with an unfavorable outcome were 62. The mean age of the patients was 71 years, and 48.5% were male. Approximately 61.2% of patients (63/103) had atrial fibrillation, 28.2% (29/103) had coronary heart disease, 13.6% (14/103) had valvular heart disease, 56.3% (58/103) had hypertension, 17.5% (18/103) hyperlipidemia, 25.2% (26/103) had diabetes, and 24.2% (25/103) had a previous stroke. Compared to patients with favorable functional outcome, those with unfavorable outcome were significantly older, had a higher proportion of previous stroke, higher baseline NIHSS, 24-h NIHSS, ΔNIHSS, percent ΔNIHSS score, higher rates of post-procedural ICH, and a significantly lower proportion of mTICI 3 recanalization. No significant differences were observed between the two groups regarding sex, BMI, smoking, alcohol consumption, hypertension, diabetes, hyperlipidemia, atrial fibrillation, coronary heart disease, valvular heart disease, occlusion site, procedure duration, OPT, PRT, bridging thrombolysis, pre-stroke mRS, ASTIN/SIR score and CT-ASPECTS (Table 1).

**Table 1:** Clinical characteristics of the study cohort.

Characteristics	mRS 0–2 (*n* = 41)	mRS 3–6 (*n* = 62)	*t*/*χ*^2^/*Z*	*p* value
Age, years, mean ± SD	66.0 (57.5, 72.5)	72.5 (67.0, 80.0)	−3.905	0.000
Male, *n* (%)	22 (53.7)	28 (45.2)	0.713	0.398
BMI	24.84 ± 2.91	24.00 ± 3.91	1.075	0.285
History				
Smoke, *n* (%)	10 (24.4)	11 (17.7)	0.672	0.412
Alcohol consumption, *n* (%)	10 (24.4)	9 (14.5)	1.599	0.206
Hypertension, *n* (%)	20 (48.8)	38 (61.3)	1.570	0.210
Hyperlipidemia, *n* (%)	5 (12.2)	13 (21.0)	1.317	0.298
Diabetes mellitus, *n* (%)	9 (22.0)	17 (27.4)	0.391	0.532
Atrial fibrillation, *n* (%)	24 (58.5)	39 (62.9)	0.198	0.656
Previous stroke, *n* (%)	5 (12.2)	20 (32.3)	5.405	0.020
Coronary heart disease, *n* (%)	11 (26.8)	18 (29.0)	0.059	0.808
Valvular heart disease, *n* (%)	7 (17.1)	7 (11.3)	0.703	0.402
Embolization site			4.249	0.236
M1 segment of MCA	27 (65.9)	32 (51.6)		
M2 segment of MCA	5 (12.2)	14 (22.6)		
ICA	8 (19.5)	16 (25.8)		
ACA	1 (2.4)	0 (0.0)		
OPT, minute, median (IQR)	252.0 (178.3, 342.3)	240.0 (181.5, 317.0)	−0.336	0.737
PRT, minute, median (IQR)	61.0 (47.5, 91.5)	76.0 (55.5, 97.3)	−1.445	0.148
‌Bridging thrombolysis, *n* (%)	16 (42.1%)	22 (57.9%)	0.133	0.751
Thrombectomy, count, median (IQR)	2.0 (1.0, 2.0)	2.0 (1.0, 3.0)	−1.574	0.116
mRS pre-stroke, score, median (IQR)	0.0 (0.0, 0.0)	0.0 (0.0, 0.0)	−1.591	0.112
NIHSS score, median (IQR)				
Baseline NIHSS	15.0 (10.3, 18.0)	18.0 (14.0, 21.0)	−3.279	0.001
24-h NIHSS	7.0 (2.3, 11.8)	18.0 (12.0, 24.5)	−5.929	0.000
ΔNIHSS	7.0 (1.0, 10.0)	0.0 (−3.0, 5.0)	−3.887	0.000
Percent ΔNIHSS, %	53.08 (19.2, 80.9)	0.0 (−14.9, 31.2)	−4.762	0.000
ASTIN/SIR, score, median (IQR)	2.0 (2.0, 3.0)	3.0 (1.0, 3.0)	−0.570	0.569
mTICI, *n* (%)			7.351	0.007
2b	12 (25.5)	35 (74.5)		
3	29 (51.8)	27 (48.2)		
CT-ASPECTS	7.0 (5.0, 9.0)	7.0 (4.0, 8.0)	−1.276	0.202
ICH	15.0 (27.8)	39.0 (72.2)	6.854	0.009

Continuous variables are expressed as mean ± SD or median (interquartile range). Categorical variables are expressed as frequency (percentage). IQR, interquartile range; SD, standard deviation; OPT, onset to puncture time; PRT, puncture to reperfusion time; mRS, modified Rankin Scale; NIHSS, National Institute of Health Stroke Scale; MCA, Middle Cerebral Artery; ICA, Internal Carotid Artery; ACA, Anterior Cerebral Artery; mTICI, modified Thrombolysis in Cerebral Infarction; CT-ASPECTS, Alberta Stroke Program Early CT Score; ICH, intracranial hemorrhage.

### Multivariate analysis of unfavorable outcome

To evaluate the independent predictive value of NIHSS-related metric, four multivariate logistic regression models were constructed (Table 2), the results showed that baseline NIHSS, 24-h NIHSS, ΔNIHSS, and percent ΔNIHSS were all independent factors influencing 90-day unfavorable functional outcome. Baseline NIHSS (OR, 1.113 [95%CI, 1.018–1.218]) and 24-h NIHSS score (OR, 1.189 [95% CI, 1.087–1.301]) showed a significant positive association with 90-day unfavorable functional outcome, whereas ΔNIHSS score (OR, 0.928 [95% CI, 0.866–0.993]) and the percent ΔNIHSS score (OR, 0.984 [95% CI, 0.971–0.996]) showed significant inverse associations with 90-day favorable functional outcome after adjusting for age, history of stroke, mTICI3 (yes = 1, no = 0) and ICH. We constructed an additional multivariate logistic regression model that included, alongside the 24-h NIHSS, all prespecified atherosclerotic risk factors: hypertension, Diabetes mellitus, coronary heart disease, smoking status, and hyperlipidemia. In this fully adjusted model, the 24-h NIHSS remained a strong independent predictor of 90-day unfavorable outcome (OR, 1.207; 95% CI, 1.094–1.331; *p* < 0.001), with an AUC of 0.850 (95% CI, 0.774–0.925) (Supplementary Table 1).

**Table 2:** Multivariate analysis of unfavorable outcome.

Variables	Model 1		Model 2		Model 3		Model 4	
	OR (95%CI)	*p*-value	OR (95%CI)	*p*-value	OR (95%CI)	*p*-value	OR (95%CI)	*p*-value
Age	1.055 (1.008–1.105)	0.022	1.039 (0.989–1.092)	0.127	1.064 (1.016–1.115)	0.009	1.054 (1.006–1.105)	0.027
Previous stroke	1.739 (0.505–5.982)	0.380	2.034 (0.535–7.730)	0.297	2.203 (0.654–7.425)	0.202	2.05 (0.606–6.931)	0.248
Baseline NIHSS	1.113 (1.018–1.218)	0.019						
24-hour NIHSS			1.189 (1.087–1.301)	0.000				
ΔNIHSS					0.928 (0.866–0.993)	0.031		
PercentΔNIHSS							0.984 (0.971–0.996)	0.011
mTICI 3	0.276 (0.104–0.734)	0.01	0.447 (0.154–1.295)	0.138	0.382 (0.144–1.013)	0.053	0.448 (0.165–1.222)	0.117
ICH	1.966 (0.762–5.072)	0.162	1.327 (0.454–3.886)	0.605	1.986 (0.760–5.188)	0.161	1.709 (0.634–4.608)	0.290

NIHSS, National Institute of Health Stroke Scale; mTICI, modified Thrombolysis in Cerebral Infarction; ICH, intracranial hemorrhage.

### Receiver operating characteristic curves analysis

**Figure 1:**
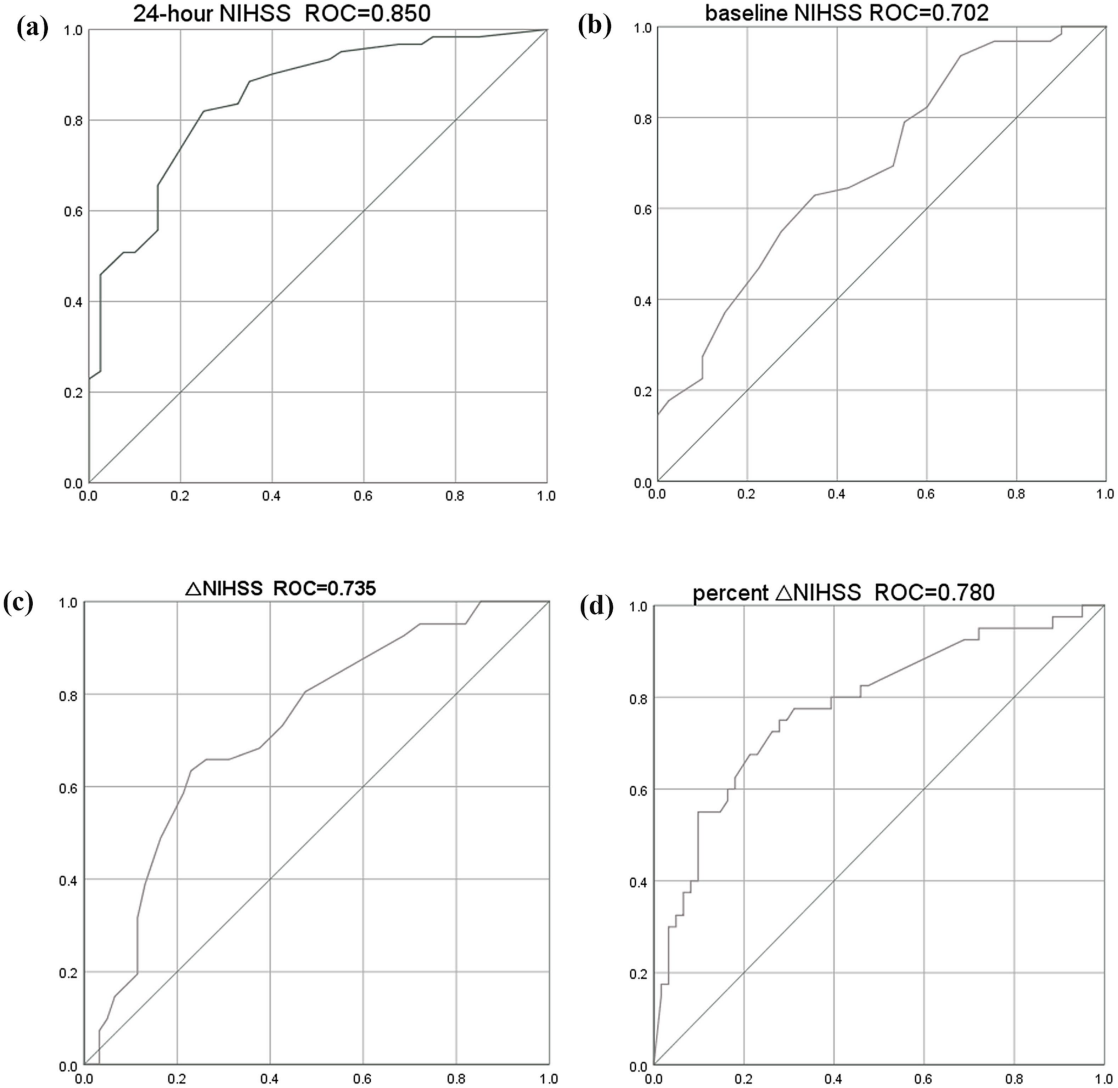
Receiver operating characteristic curves for predicting 90-day unfavorable functional outcome. **(a)** 24-h NIHSS score; **(b)** Baseline NIHSS score; **(c)** -ΔNIHSS score (negative value of ΔNIHSS); **(d)** -Percent ΔNIHSS score (negative value of percent ΔNIHSS). The negative transformation for ΔNIHSS and percent ΔNIHSS was applied to align the direction of association with unfavorable outcome for comparative purposes.

The 24-h NIHSS score (AUC, 0.850 [95% CI, 0.774–0.925]) had the highest discriminative ability to predict unfavorable functional outcome (mRS score 3–6) at 90-day compared with baseline NIHSS (AUC, 0.702 [95% CI, 0.599–0.806]), ΔNIHSS (AUC, 0.735 [95% CI, 0.636–0.834]), and percent ΔNIHSS (AUC, 0.780 [95% CI, 0.686–0.875]) scores (Figure 1). The Youden index identified a 24-h NIHSS score of ≥12 as the threshold for predicting an mRS score of 3–6, demonstrating good sensitivity (82.2%) and specificity (75.6%).

### Delong’s test for model comparison

To further validate the superiority of the 24-h NIHSS, DeLong’s test was used for pairwise comparison of the predictive performance of the four NIHSS scores, and to compare the predictive performance of the 24-h NIHSS alone versus a combined model incorporating all four NIHSS scores for 90-day unfavorable outcome. The results showed that the predictive performance of 24-h NIHSS was significantly higher than that of baseline NIHSS, ΔNIHSS, and percent ΔNIHSS. The combined model of all four NIHSS scores (AUC, 0.852 [95% CI, 0.776–0.927]) showed no significant difference in predictive ability compared to 24-h NIHSS (AUC, 0.850 [95% CI, 0.774–0.925]) (*p* = 0.656), indicating that 24-h NIHSS has strong predictive power for 90-day unfavorable outcome (Table 3).

**Table 3:** DeLong’s test for model comparison.

Comparison model	*z* value	*p* value	Difference in AUC	SE (*D*)	95%CI
BaselineNIHSS-24-h NIHSS	−2.815	0.005	−0.148	0.300	−0.250 to −0.045
BaselineNIHSS-ΔNIHSS	−0.388	0.698	−0.033	0.324	−0.197 to 0.132
BaselineNIHSS-percent ΔNIHSS	−1.018	0.309	−0.078	0.320	−0.229 to 0.720
24-h NIHSS-ΔNIHSS	2.891	0.004	0.115	0.294	0.037 to 0.193
24-h NIHSS-percent ΔNIHSS	2.044	0.041	0.007	0.290	0.003 to 0.136
ΔNIHSS-percent ΔNIHSS	−2.451	0.014	−0.045	0.307	−0.082 to −0.009
24-h NIHSS-combined model	−0.445	0.656	−0.002	0.272	−0.011 to 0.007

Pairwise comparisons of the predictive value of baseline NIHSS, ΔNIHSS, percent ΔNIHSS and 24-h NIHSS for 90-day unfavorable outcome.

### The distribution map of functional outcome

**Figure 2:**
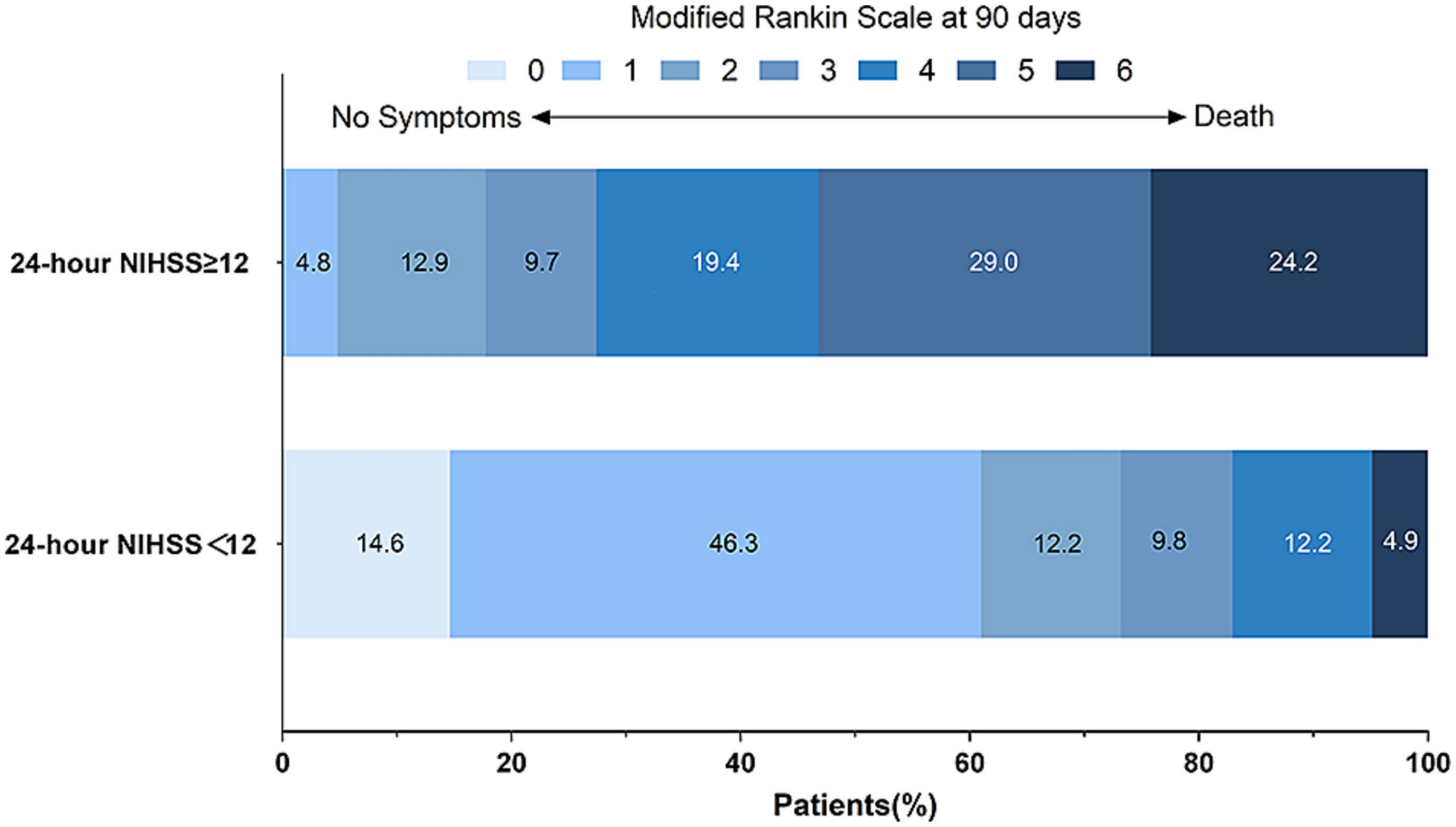
Distribution of 90-day modified Rankin scale scores stratified by the 24-h NIHSS cutoff (≥12 vs. <12). The proportion of patients with severe disability or death (mRS 5–6) was significantly higher in the group with 24-h NIHSS score ≥12.

Based on the optimal cutoff value (12 points) determined by ROC analysis, patients were divided into a 24-h NIHSS score <12 group (*n* = 42) and a ≥ 12 group (*n* = 61). The distribution map of functional outcome (Figure 2) clearly demonstrates significant differences in the distribution of 90-day mRS scores between the two groups. Among patients with 24-h NIHSS ≥12, the proportion of mRS 5–6 (death and severe disability) was significantly higher, while among patients with 24-h NIHSS score <12, the proportion of mRS 0–2 (functional independence) was dominant. This visually confirms the clinical utility of the 24-h NIHSS.

## Discussion

This retrospective study of 103 cardioembolic stroke patients with ACO undergoing EVT found that baseline NIHSS, 24-h NIHSS, ΔNIHSS, and percent ΔNIHSS were all independent predictors of 90-day unfavorable functional outcome. Among these, the 24-h NIHSS demonstrated the strongest predictive ability. The Youden index identified a 24-h NIHSS score of ≥12 as the threshold for predicting an mRS score of 3–6 at 90-day after EVT, demonstrating good sensitivity (82.2%) and specificity (75.6%). Furthermore, rigorous statistical analysis (DeLong’s test) showed that combining baseline NIHSS, ΔNIHSS, and percent ΔNIHSS did not significantly improve the predictive performance of the 24-h NIHSS. This suggests that the 24-h NIHSS may already integrate the effective prognostic information contained in other NIHSS-derived metrics, making it a concise and powerful early prediction tool. These findings are consistent with recent studies demonstrating that the 24-h NIHSS outperforms other NIHSS-based metrics in EVT-treated populations (18).

High NIHSS scores are associated with unfavorable outcome; therefore, NIHSS is often used as a predictive indicator for prognosis in acute cerebral infarction (8, 21, 22). A study on predictors of effectiveness of EVT for large vessel occlusion stroke showed that among patients with severe stroke (NIHSS ≥20), approximately 66% did not achieve a favorable outcome despite successful recanalization (23). Furthermore, high NIHSS has been reported as an independent predictor of futile recanalization (24). While some studies suggest that dynamic measures of neurological improvement, such as the absolute (ΔNIHSS) or relative (percent ΔNIHSS) change in NIHSS, may offer superior prediction over a single baseline assessment (18, 25), the comparative prognostic value of a static assessment at the critical 24-h after EVT has been less clear, especially in homogeneous patient populations.

The superior predictive value of the 24-h NIHSS for 90-day functional outcome, compared to baseline NIHSS or its change (ΔNIHSS), has been established across multiple cohorts of EVT-treated patients. This holds true for anterior circulation strokes (7, 18) as well as posterior circulation occlusions (26), including within cardioembolic subgroups (27). However, existing evidence originates from studies encompassing heterogeneous populations with mixed stroke etiologies and, crucially, variable rates of successful recanalization (mTICI ≥2b rates: 80.6–93.2%) (7, 18, 26, 27). The inclusion of patients with failed or incomplete recanalization may confound the observed relationship between early neurological status and final outcome. To address this limitation, our study specifically focused on a homogeneous cohort of cardioembolic stroke patients with ACO, thereby eliminating confounding from other stroke mechanisms. We exclusively included EVT-treated patients who all achieved successful reperfusion (mTICI ≥2b). This design isolates the prognostic role of early post-treatment neurological severity from the dominant confounder of recanalization failure. Notably, our assessment of “early” neurological status was timed at 24 h after the EVT procedure, not from symptom onset, directly incorporating the procedural effect into the evaluated metric. Our findings are further supported by a study that restricted analysis to an even more selective population—patients who achieved complete recanalization (mTICI 2c/3). Neuberger U et al. demonstrated that in anterior circulation stroke patients with complete recanalization after EVT, 24-h NIHSS score of ≤5 accurately predicted favorable long-term outcome, with an AUC of 0.88 (95% CI: 0.84–0.92) (28). Notably, the optimal cutoff value in their complete recanalization cohort (≤5) is more stringent than the threshold we identified in our successful reperfusion cohort (≥12 for unfavorable outcome), which likely reflects the better baseline prognosis of patients achieving mTICI 3 recanalization. Taken together, these complementary findings suggest that the 24-h NIHSS serves as a robust prognostic marker across the entire spectrum of successful recanalization, with cutoff values that can be calibrated according to the achieved recanalization grade.

Beyond our specific cohort, the prognostic value of the 24-h NIHSS has been consistently validated across multiple independent cohorts and different vascular territories. In a study of 108 anterior circulation stroke patients undergoing EVT, Katano et al. demonstrated that a 24-h NIHSS <10 predicted favorable outcome with an AUC of 0.89, significantly outperforming baseline NIHSS (7). Extending these findings to the posterior circulation, the same research group recently reported that in 164 vertebrobasilar occlusion patients, a 24-h NIHSS ≤10 predicted good outcome (AUC 0.86) while a score ≥15 predicted 90-day mortality (AUC 0.81) (29). A large-scale German registry study of 1,268 isolated MCA-M2 occlusions further confirmed that 24-h NIHSS ≤8 predicts favorable outcome with an AUC of 0.85 (95% CI: 0.83–0.87) (30). Beyond its predictive utility, the 24-h NIHSS has been proposed as a surrogate outcome measure in acute stroke trials. A pooled analysis of 7 randomized EVT trials involving 1,720 patients demonstrated that 24-h NIHSS mediated the association between EVT and 90-day mRS, meeting criteria for a useful surrogate endpoint (31). This methodological validation further underscores the clinical relevance of our findings. Importantly, A large analysis from the ENCHANTED study of 4,496 thrombolysis patients revealed that cardioembolic stroke subtype was an independent predictor of both early and delayed neurological deterioration (both *p* ≤ 0.01) (32). This mechanistic insight provides a compelling explanation for why the 24-h NIHSS exhibits particularly strong predictive value in our cardioembolic cohort—these patients are inherently at higher risk of early neurological changes due to the friable nature of cardiac emboli and their propensity for early reocclusion or hemorrhagic transformation. Consequently, the 24-h NIHSS serves as an ideal composite marker capturing the net effect of initial severity, recanalization success, and early complications in this vulnerable population.

Consistent with these external validations, the 24-h NIHSS emerged as the strongest predictor even within our rigorously defined cohort. This finding underscores that the post-procedural NIHSS score is not merely a static measure but a composite endpoint that inherently encapsulates the net effect of initial stroke severity, the efficacy of recanalization, and the early response to treatment—including complications such as hemorrhagic transformation. Therefore, the 24-h NIHSS can reflect the impact of numerous other parameters that influence long-term outcome in stroke patients. Moreover, some patients who achieve recanalization exhibit “delayed neurological response” (delayed or no response to recanalization for several hours) and may achieve significant neurological recovery at a later stage (33). In contrast, baseline NIHSS represents only the starting point of injury. While ΔNIHSS and percent ΔNIHSS are good predictors of neurological improvement, they may not be the optimal predictors of final clinical outcome, as they quantify the amount of change but may overlook the prognostic significance of the absolute level of neurological deficit. For example, a patient improving from a baseline of 25 to 15 (ΔNIHSS = 10) may still have a worse prognosis implied by their 24-h score of 15 (severe disability) compared to a patient improving from 15 to 5 (ΔNIHSS = 10). This limitation of ΔNIHSS can be explained statistically. The calculation of ΔNIHSS implicitly assumes a 1:1 relationship between baseline and 24-h NIHSS, an assumption that is rarely met due to the non-linear nature of the scale (34). As demonstrated by Mistry et al. (34) analysis of covariance (ANCOVA)—with follow-up NIHSS as the dependent variable and baseline NIHSS as a covariate—is statistically more efficient than analyzing raw change scores because it allows the data to estimate the actual relationship rather than assuming a fixed 1:1 effect. Applying this ANCOVA framework to our cohort (Supplementary Table 2), we found that the estimated coefficient for baseline NIHSS was 0.462 (95% CI: 0.233–0.691), significantly less than 1 (*p* < 0.001). This empirical evidence supports that the poor predictive performance of ΔNIHSS in our study is at least partially attributable to the violation of its underlying statistical assumption.

## Conclusion

This study is the first to demonstrate that the 24-h NIHSS score is the strongest predictor in a homogeneous cohort of cardioembolic stroke patients with ACO patients who achieved successful recanalization after EVT. 24-h NIHSS score ≥12 points is the optimal cutoff for predicting 90-day functional unfavorable outcome. This metric is readily available in routine clinical practice and can serve as an effective tool for assessing 90-day post-procedural functional prognosis, aiding in patient management and clinical decision-making. This study has several limitations, including its single-center retrospective design, relatively limited sample size (which may affect statistical power), and lack of external validation. Furthermore, our findings may not be generalizable to patients with failed recanalization (mTICI 0–2a). Future multicenter studies with larger sample sizes and broader inclusion of recanalization outcomes are needed to validate the prognostic utility of the 24-h NIHSS in the EVT-treated population.

## Data Availability

The raw data supporting the conclusions of this article will be made available by the authors, without undue reservation.
