# Supplementary material for: 24-h NIHSS score is the strongest prognostic predictor of 90-day outcome in cardioembolic stroke patients with anterior circulation occlusion after endovascular thrombectomy
Source: Front Neurol. 2026 May 13;17:1821974. doi: 10.3389/fneur.2026.1821974 (PMC13215112; doi:10.3389/fneur.2026.1821974)
Supplement: Supplementary file 1 [file Table_1.docx]

Supplementary Material Table 1

**Multivariate regression model for correcting risk factors of atherosclerosis**

| Variables |  | OR(95％CI) | P-value |
| --- | --- | --- | --- |
| Age |  | 1.046（0.991-1.104） | 0.104 |
| Smoke |  | 2.379（0.598-9.459） | 0.219 |
| Hypertension |  | 1.420（0.416-4.850） | 0.576 |
| Diabetes mellitus |  | 0.667(0.179-2.562) | 0.566 |
| Hyperlipidemia |  | 0.758(0.168-3.429) | 0.719 |
| Coronary heart disease |  | 1.481(0.443-4.950) | 0.524 |
| Previous stroke |  | 2.052(0.476-8.854) | 0.335 |
| 24h NIHSS |  | 1.207(1.094-1.331) | 0.000 |
| mTICI 3 |  | 0.481(0.160-1.445) | 0.192 |
| ICH |  | 1.455（0.464-4.563） | 0.520 |

Supplementary Material Table 2

**Analysis of Covariance (ANCOVA) for 24-Hour NIHSS**

| Variables | B | 95％CI | P-value |
| --- | --- | --- | --- |
| Baseline NIHSS | 0.462 | 0.233–0.691 | 0.000 |
| 90-day outcome (favorable vs. unfavorable) | 8.660 | 5.460–11.860 | 0.000 |

Dependent variable: 24-hour NIHSS. Model R² = 0.413, adjusted R² = 0.401.
